# Revised short screening version of the attachment questionnaire for couples from the German general population

**DOI:** 10.1371/journal.pone.0230864

**Published:** 2020-04-02

**Authors:** Katja Petrowski, Elmar Brähler, Thomas Suslow, Markus Zenger

**Affiliations:** 1 Medical Psychology and Medical Sociology, University Medical Center of the Johannes Gutenberg-University, Mainz, Germany; 2 Department of Mental Health University Medical Center of university of Leipzig, Germany, Leipzig, Germany; 3 Faculty of Applied Human Studies, University of Applied Sciences Magdeburg and Stendal, Stendal, Germany; 4 Integrated Research and Treatment Center (IFB) AdiposityDiseases—Behavioral Medicine, Psychosomatic Medicine and Psychotherapy, University of Leipzig Medical Center, Leipzig, Germany; Iranian Institute for Health Sciences Research, ISLAMIC REPUBLIC OF IRAN

## Abstract

In order to ensure the concentration, compliance and motivation of the participants, a short version of 12 items was extracted from the “Experiences in Close Relationships Questionnaire (ECR)”. Even though this short English version shows equally good validity and reliability as the long version, there have been no representative norm values and psychometric characteristics available for the short version in German. Therefore, the German 12-item ECR was implemented in a representative sample of 1,127 males and 1,237 females (mean age *M* = 49.93; *SD* = 12.31) from the general public (N = 2,364). The reliability values of the German 12-item ECR in the representative sample are not as good as the long version with the 36- items version (Alpha = .54–72), and the 12-item ECR factorial structure failed to show the factorial validity. Since the EFA revealed that only half the items loaded on the expected factors, an even shorter form with six items was construed and tested psychometrically. Even though the item numbers were reduced, the reliability values of the German 6-item ECR improved and were as good as the long version with 36 items (Alpha = .73–90). Furthermore, factorial validity could be shown by CFA (CFI = .981, SRMR = .038, RMSEA = .079, TLI = .964) with scalar invariance across gender and age. In sum, the 6-item ECR is a reliable and factorial scalar attachment questionnaire. Due to its shortness, it is applicable to different research fields. However, reference data from a clinical sample are still missing.

## Introduction

The personal attachment between two closely related individuals is an extensively researched field [1, 2] which has already engendered various concepts and models [3, 4, 5]. John Bowlby [3] formulated the terms for the three attachment styles: secure, insecure-avoidant, and insecure-anxious-ambivalent. Thereby, a secure attachment style means that the individual will seek help from a significant other (reference person) in stress situations, whereas an insecure individual will not seek any support from a reference person and an anxious individual will make inconsistent, insecure attempts to ensure the support of the reference person [6].

Bartholomew and Horowitz [5, 7] postulated two orthogonal dimensions resulting in four attachment styles. One dimension includes the self-concept, which can be specified in a spectrum from positive to negative, while the other dimension measures the positive or rather the negative image in respect to reference persons. Securely attached individuals show a positive manifestation in both dimensions, while insecurely attached individuals have a negative attitude toward themselves, yet a positive one toward others. Anxiously attached individuals strike out negatively in both dimensions, and avoidant ones show a positive attitude toward themselves as well as a negative one toward other individuals [8].

In order to measure this concept, the “Experiences in Close Relationships Questionnaire” (ECR; [10]) was developed and later translated into German (ECR-G; [9, 10]). The ECR questionnaire is a multi-item scale in respect to partnerships. Based on a major components analysis, the factors avoidance and anxiety could be identified. For each of these factors, a scale of 18 items was developed with a total of 36 items for the dimensions anxiety and avoidance in relationships, which was matched to the four attachment styles according to Bartholomew and Horowitz [5].

Regarding the adaptation of the ECR into a German version, the items were evaluated on a five-step instead of a seven-step Likert scale from “does not apply at all”to “applies”as well as to “neither/nor” in the center to guarantee greater clarity and to avoid making an excessive demand on the test persons [9]. The ECR-G was tested in two different samples, one with students and the other a clinical sample including outpatient psychotherapy patients [9]. The ECR-G scales regarding age and duration of the relationship showed low correlations, nevertheless, they became significant for three of the four investigated connections in the student sample. In this sample, avoidance grew with age while anxiety diminished. Furthermore, anxiety also diminished with the duration of the relationship. In the clinical sample, no such connections could be found.

The two-factorial structure of the ECR could also be ascertained in the German adaptation ECR-G by establishing that all 36 items showed a high load on the respective factor as well as a low one on the respective other factor. The two scale dimensions showed a non-significant positive connection and can thus be termed as independent [9, 11]. Furthermore, it could be shown that Cronbach‘s Alpha for scale avoidance was at .89 and at .57 for the anxiety scale [12]. These high values for the internal consistency were also confirmed in other studies [13, 14].

In addition, the ECR-G was compared with other scales for testing partnerships and attachment in order to obtain sufficient validation. So it could be shown that avoidance correlated negatively with stability of the relationship and sexual satisfaction. High construct validity was given. Concerning personality characteristics, a positive connection was found between the anxiety scale and neuroticism [11]. Furthermore, there existed a strong connection between narcissism and a high manifestation on the anxiety scale [15]. Additionally, Doering et al. [16] were able to show that the anxiety as well as the avoidance scale correlated negatively with self-concept and need to control as well as positively with social stress. Stress perception also correlated negatively with the anxiety scale [11].

Since the length of the ECR-G questionnaire (36 items) might be problematic in some research applications, the original authors of the English version developed a 12-item ECR short version [17]. The confirmatory factor analyses indicated two factors: anxiety and avoidance. This short version showed equally good validity and reliability as the long version [17]. Also, for the German ECR-G, a 12- item version (ECR-S12) was developed to ensure broad utilization in surveys and high compliance. However, for this short version (ECR-S12), no representative norm values and psychometric characteristics were available. Therefore, the 12-item ECR-S12 was implemented in a representative sample from the general population.

### Method

The present study was part of a national representative survey of the general population of Germany. Data were collected in 2011 by an independent institute for opinion and social research (USUMA, Berlin). The criteria for inclusion were a minimum age of ≥14 and sufficient ability to understand the written German language. After a socio-demographic interview, the participants completed self-report questionnaires regarding physical and psychological symptoms in the presence of (but without any interference from) the interviewer. A random-route sampling procedure with 258 sample points revealed that 4,386 households needed to be contacted for the study. Of these, 4,327 households proved eligible for participation. The selection of the target individuals within the households was carried out according to the Kish selection grid [18]. In total, 2,555 individuals took part in the study (participation rate 59%). Subjects younger than 18 years of age (n = 70) and subjects with missing data in at least one of the items (n = 121) were excluded from the analysis. Thus, the final sample consisted of 2,364 individuals. This study, including the consent procedure, was approved by the institutional ethics review board of the University of Leipzig (Az 063-14-10032014). Furthermore, the study adhered to ICH-GCP-guidelines along with the ICC/ESOMAR International Code of Marketing and Social Research Practice. All participants were informed of the study procedures, data collection and anonymization of all personal data. All the participants provided verbal informed consent according to German law, which was documented by the interviewer before starting the survey.

### Statistical analyses

The factorial structure of the Experiences in Close Relationship Scale—Short Form (ECR-S12) by Wei et al. [17] was tested using the confirmatory factor analysis (CFA), calculated with AMOS© 20. All the models were tested using covariance matrices, and each model was estimated with the maximum likelihood method approach. All the models were compared to each other on the basis of the following model fit indices: the minimum discrepancy divided by its degrees of freedom (CMIN/DF); the comparative-fit-index (CFI); standardized root mean square residual (SRMR); the root mean square error of approximation (RMSEA); the Tucker-Lewis Index (TLI), and the Bayesian Information Criterion (BIC). For a good model fit, the ratio CMIN/DF should be as small as possible [19]; values of TLI and CFI close to 0.95 or higher are indicative of a good or at least acceptable (>.90) model fit. Furthermore, RMSEA should be 0.08, and SRMR should be 0.05 or smaller. The BIC is a descriptive indicator of poor fit and allows for comparisons between two models; the model with the lower BIC should be preferred [19].

Additional analyses were conducted to test the invariance of the model across gender and age using multi-group CFA [20]. After testing the factorial structure in each subgroup, measurement invariance was tested in three steps first using the configural model (no constraints), followed by a metric invariant model (with unstandardized item loadings constrained to be equal across groups) and a scalar invariant model (with unstandardized item loadings and unstandardized item intercepts simultaneously constrained to be equal across groups). Based on the hierarchy of these nested and increasingly restrictive models, the models were then compared to each other. Since the χ2 statistic has often been criticized for its sensitivity to sample size, the main focus was placed on the differences of the ΔCFI and the ΔRMSEA. Values smaller than .01 indicate the invariance of the models [21]. To avoid the potential problem of selecting a marker variable that is possibly not invariant, the variance of each latent variable was fixed to 1.0 (and the mean was fixed to 0.0) for scaling purposes [22].

The remaining statistical analyses were conducted using the IBM SPSS© version 20. In a first step, the original factor structure of the ECR-S12 was tested by CFA. According to the great misfit between data and the original two-factor model of the ECR-S12 (see Table 4), an exploratory factor analysis (EFA) was employed after a random split of the study sample. Both subsamples did not differ significantly with regard to gender and age. A principal axis factors method with varimax rotation was conducted with subsample 1 (*N* = 1,104). Subsequently, CFAs were conducted with subsample 2 (*N* = 1,260).

## Results

Sociodemographic characteristics are presented in Table 1.

**Table 1:** Sociodemographic characteristics of the study population.

	Total *N* = 2,364	Men *N* = 1,127	Women *N* = 1,237
	*M (SD)*	*M (SD)*	*M (SD)*
**Age, years**	49.93 (17.31)	49.91 (17.39)	49.95 (17.25)
**Age range**	18–97	18–97	18–90
**Age groups**	***N (%)***	***N (%)***	***N (%)***
18–29 years	362 (15.3)	173 (15.4)	189 (15.3)
30–39 years	334 (14.1)	166 (14.7)	168 (13.6)
40–49 years	475 (20.1)	220 (19.5)	255 (20.6)
50–59 years	436 (18.4)	204 (18.1)	232 (18.8)
60–69 years	380 (16.1)	183 (16.2)	197 (15.9)
≥70 years	377 (15.9)	181 (16.1)	196 (15.8)
**Relationship status**			
Married/ Living together	1,225 (51.8)	613 (54.4)	612 (49.5)
Married/ separated	37 (1.6)	9 (0.8)	28 (2.3)
Unmarried	557 (23.6)	312 (27.7)	245 (19.8)
Divorced	285 (12.1)	119 (10.6)	166 (13.4)
Widowed	260 (11.0)	74 (6.6)	186 (15.0)
**Living in partnership**			
Yes	1,439 (60.9%)	721 (64.0%)	718 (58.0%)
No	925 (39.1%)	406 (36.0%)	519 (42.0%)
**Education**			
≤ 8 years	991 (41.9%)	461 (40.9%)	530 (42.8%)
9–11 years	995 (40.4%)	430 (38.2%)	525 (42.4%)
≥ 12 years	400 (16.9%)	226 (20.1%)	174 (14.1%)
School student	8 (0.3%)	3 (0.3%)	5 (0.4%)
Missing	10 (0.4%)	7 (0.6%)	3 (0.2%)
**Employment status**			
Education/Training	200 (8.0)	77 (6.8)	57 (4.6)
Working	1,293 (51.6)	616 (54.7)	544 (44.0)
Unemployed/Working <15h per week	159 (6.3)	94 (8.3)	145 (11.7)
Housewife/House husband	130 (5.2)	4 (0.4)	114 (9.2)
Retired	726 (28.9)	336 (29.8)	374 (30.2)
Missing	3 (0.1%)		3 (0.2%)

### Reliability of the ECR-S12

The Cronbach’s Alpha for the anxiety scale was = .538, and for the avoidance scale it was *Alpha* = .724.

### Exploratory factor analysis (EFA)

The results of the EFA indicated a two- factor solution using the Kaiser Guttman criterion with eigenvalues of 3.66 and 3.25, accounting for 30% and 27% of the variance, respectively. On the one hand, this is within the average results of the meta-analysis by Peterson (containing 803 studies), on the other hand, it is a rather low percentage compared to the results of 50% variance by random data [23]. Factor loadings ranged between .71 and .79 on the first factor (including items 1, 2, 3, 4, 5, 8) and between .55 and .89 on the second factor (including items 6, 7, 9, 10, 11, 12). Factor loadings are presented in Table 2. A comparison with the original ECR-S shows that only half the items loaded on their expected factor.

**Table 2:** Factor loadings derived from EFA using principal axis factors method (rotated component matrix).

Item No.		Rotated solution	
	Expected Factor*	Component 1	Component 2
1	1	**.731**	-.055
2	2	**.708**	-.115
3	2	**.747**	.131
4	1	**.782**	.028
5	2	**.792**	.115
6	1	.287	**-.665**
7	1	.081	**.550**
8	1	**.738**	-.072
9	2	.109	**.863**
10	1	.385	**-.442**
11	2	.068	**.892**
12	2	.033	**.863**

*According to original ECR-S: 1 = anxiety factor, 2 = avoidance factor

### Confirmatory factor analysis (CFA)

According to the results of the EFA, this model was tested by CFA in subsample 2. The results, as shown in Table 3, indicate a much better model fit compared to the initially tested original two-factor structure of the ECR-S12 but a still unacceptable fit between the model and the data, according to the recommended model fit indices.

**Table 3:** Summary of fit indices of different factor models.

Model	χ ^2^ (df)	CMIN/DF	CFI	SRMR	RMSEA (CI)	TLI	BIC
Original two-factor model	7170.728 (53)	135.297	.445	.210	.233 (.228-.237)	.309	7,366.179
Two-factor model according to EFA results	804.349 (53)	15.176	.885	.104	.106 (.100-.113)	.856	982.820
Two-factor model short form ECR-S6	71.392 (8)	8.924	.981	.038	.079 (.063-.097)	.964	164.197

df = degrees of freedom; CMIN/DF = minimum discrepancy, divided by its degrees of freedom; CFI = comparative-fit-index; SRMR = standardized root mean square residual; RMSEA (CI) = root mean square error of approximation (confidence interval); TLI = Tucker-Lewis Index; BIC = Bayesian Information Criterion

Furthermore, descriptive item characteristics (please see Table 4) revealed some weaknesses of the ECR-S12 items with regard to the corrected item-total correlation as well as the difficulty index.

**Table 4:** Characteristics of the ECR-S12 items.

Item/Scale	*M*(*SD*)	Skewness	Kurtosis	*P*	*r*_*it*_
Item 1 ^a^	2.36 (1.63)	1.03	0.07	.23	.54
Item 2 ^b^	2.59 (1.71)	0.83	-0.40	.26	.16
Item 3 ^b^	1.80 (1.25)	1.67	2.16	.13	.42
Item 4 ^a^	1.86 (1.27)	1.57	1.81	.14	.45
Item 5 ^b^	1.93 (1.39)	1.56	1.71	.16	.39
Item 6 ^a^	4.37 (2.01)	-0.38	-1.06	.56	.21
Item 7 ^a^	3.75 (2.09)	0.19	-1.26	.46	-.09
Item 8 ^a^	2.48 (1.66)	0.95	-0.09	.25	.48
Item 9 ^b^	3.14 (2.02)	0.13	-1.26	.36	.62
Item 10 ^a^	3.63 (2.05)	0.65	-0.85	.44	.35
Item 11 ^b^	2.91 (1.94)	0.87	-0.39	.32	.64
Item 12 ^b^	3.21 (1.94)	0.64	-0.70	.37	.57
Anxiety scale	3.07 (0.99)	0.36	<0.01		
Avoidance scale	3.09 (1.80)	0.78	-0.39		

^a^ anxiety item

^b^ avoidance item *;P*, difficulty index; *r*_*it*_, corrected item-total correlation.

### Short form ECR-S6

Therefore, the six items not loading on the expected factor of the original ECR-S12 scale were deleted, and a 6-item short form of the original ECR-S12 (including Items 1, 4, and 8 for the anxiety subscale and items 9, 11, and 12 for the avoidance subscale) was tested psychometrically. This procedure led to a short form of the ECR-S12 that also excluded all the items with a very low corrected item-total correlation as well as all but one item with a low difficulty index.

### Reliability of the short form ECR-S6

Cronbach’s alpha of the 3-item subcales of the ECR-S6 were .73 for the anxiety subscale and .90 for the avoidance subscale, thus being superior compared to the original subscales (.54 and .73, respectively).

### Factorial validity of the short form ECR-S6

The short form ECR-S6 was then tested by CFA for factorial validity. The results are presented in Table 5. Model fit indices indicate a good fit between the data and the model, and the BIC value indicates that this model fits much better to the data than all other models tested before.

**Table 5:** Summary of fit indices of different factor models.

Model	χ ^2^ (df)	CMIN/DF	CFI	SRMR	RMSEA (CI)	TLI	BIC
Original two-factor model	7170.728 (53)	135.297	.445	.210	.233 (.228-.237)	.309	7,366.179
Two-factor model according to EFA results	804.349 (53)	15.176	.885	.104	.106 (.100-.113)	.856	982.820
Two-factor model short form ECR-S6	71.392 (8)	8.924	.981	.038	.079 (.063-.097)	.964	164.197

df = degrees of freedom; CMIN/DF = minimum discrepancy, divided by its degrees of freedom; CFI = comparative-fit-index; SRMR = standardized root mean square residual; RMSEA (CI) = root mean square error of approximation (confidence interval); TLI = Tucker-Lewis Index; BIC = Bayesian Information Criterion

In addition to that, the measurement invariance of the short form ECR-S6 was tested across gender and six age groups. the results are shown in Table 6. The ECR-S6 could be shown to be invariant across gender and age since Δ CFI as well as Δ RMSEA were < .01.

**Table 6:** Tests for invariance across gender and age groups.

	N	χ ^2^ (df)	Δ χ ^2^	Δ p	CMIN/DF	CFI	Δ CFI	RMSEA	Δ RMSEA
**Gender**									
Men	599	37.159 (8)			4.645	.981		.078	
Women	661	37.952 (8)			4.744	.983		.075	
**Multigroup analysis**									
Configural model		75.112 (16)			4.694	.982		.054	
Metric model		85.420 (22)	10.309	.112	3.883	.981	.001	.048	.006
Scalar model		113.744 (28)	28.323	< .001	4.062	.974	.007	.049	.001
**Age**									
18–29	188	9.529 (8)			1.191	.996		.032	
30–39	167	21.535 (8)			2.692	.974		.101	
40–49	241	11.562 (8)			1.445	.993		.043	
50–59	245	35.236 (8)			4.405	.960		.118	
60–69	211	24.891 (8)			3.111	.970		.100	
≥70	208	21.494 (8)			2.687	.978		.090	
**Multi-group analysis**									
Configural model		271.608 (124)			2.190	.956		.031	
Metric model		276.436 (130)	4.829	.566	2.126	.956	< .001	.030	.001
Scalar model		282.467 (136)	6.030	.420	2.077	.956	< .001	.029	.001

df = degrees of freedom; CMIN/DF = minimum discrepancy, divided by its degrees of freedom; CFI = Comparative-Fit Index; RMSEA = root mean square error of approximation

### Convergent validity of the ECR-S6

As indicators of convergent validity of the anxiety and avoidance scale, Pearson correlation coefficients with other health- related variables were calculated. The results are presented in Table 7.

**Table 7:** Pearson correlations between ECR-S6 scales and further psychological scales.

	Anxiety scale	Avoidance scale
PHQ-9 (depression)	.32***	.14***
GAD-7 (anxiety)	.31***	.14***
Quality of life (EQ-5D)	-.11***	-.10***
Self-reported state of health (VAS)	-.11***	-.10***
Total score (PFB-K)	-.12***	-.24***
Quarreling behavior	.37***	.41***
Affectionateness	-.25***	-.36***
Similarity	-.31***	-.44***
Big Five–Extraversion	-.18***	-.17***
Big Five–Conscientiousness	-.20***	-.21***
Big Five–Agreeableness	-.07**	-.09***
Big Five–Neuroticism	.29***	.13***
Big Five–Openness	-.03 n.s.	-.15***

*** = p < .001

** = p < .01; n.s. = not significant

### Influence of sociodemografic parameters

T-tests (for gender and partnership status) and ANOVA (for the three different educational levels according to ≤ 8, 9–11 and ≥ 12 years, see Table 1) were conducted to test for mean differences in the anxiety and avoidance scale. To test the influence of age, Pearson’s correlation coefficient was calculated. Results are shown in Table 8. Effect sizes according to Cohen are reported for all mean differences.

**Table 8:** Influence of sociodemographic variables.

	(greatest) mean difference	test statistic (T; F)	p	Cohen’s d
Gender				
Anxiety scale	0.25	5.04	< .001	0.20
Avoidance scale	<0.01	0.06	.955	<0.01
Partnership				
Anxiety scale	0.21	4.10	< .001	0.17
Avoidance scale	1.25	17.96	< .001	0.74
Education				
Anxiety scale	0.03	0.13	.879	0.02
Avoidance scale	0.21	4.31	.014	0.12

Mean differences in the attachment anxiety scale were statistically significant in all but one comparison. According to Cohen’s d, all the differences were small in magnitude: women reported higher values on the attachment anxiety scale than men. Participants living with a partner reported lower scores on the attachment anxiety scale than participants not living together. No differences were found between different levels of education. Age and attachment anxiety were negatively correlated (r = -0.07; p .001), showing the minimal linear trend of attachment anxiety decreasing with increasing age.

Regarding the attachment avoidance scale, no gender differences could be observed, only the partnership status was of statistical and practical relevance. Participants living with a partner showed lower attachment avoidance scores with a medium effect size (d = 0.74). Furthermore, people with an educational level of ≤ 8 years reported significantly higher attachment avoidance levels compared to the group with 9–11 years of education, but effect sizes were small. The correlation between age and attachment avoidance was close to zero (r = .02; p = .415).

### Population-based norms

Percent rank norms (in increments of rounded 10%-percentiles) of the general population mean scores are given in Table 9 for both sub-scales.

**Table 9:** Percent rank scores and T-values for the anxiety and the avoidance scale.

Anxiety scale		Avoidance scale	
(rounded) Percent rank	Raw score	(rounded) Percent rank	Raw score
10	-	10	-
20	-	20	1.33
30	1.00	30	1.67
40	1.33	40	2.00
50	1.67	50	2.67
60	2.33	60	3.00
70	2.67	70	3.67
80	3.00	80	4.33
90	4.00	90	6.00

## Discussion

The 12- item version ECR-S12 was developed parallel to the ECR-S to ensure a broad utilization in surveys and high compliance. However, for this short version of the ECR—-S12, no representative norm values and psychometric characteristics were available as a sample from the general population .

Using a large German representative sample from the general population, the ECR-S12 with 12 items showed only a mediocre to good reliability (.54 and .73) in the present study. In contrast, the literature on the ECR-S showed reliability values of .77-.86 for anxiety and .78-.88 for avoidance in the different studies [17]. This is not as good as the long version with the 36 items (Alpha = .85–91; [24]). In addition, the present reliability values of the ECR-S12 in the representative sample are also not as good as the long version with the 36 items (Alpha = .85–91; [24]).

Nonetheless, Wei et al. [17] were able to confirm the two-factorial structure in the ECR-S in a sample with students. However, the CFA with the ECR-S12 factorial structure failed to show the factorial validity of this instrument in a representative sample from the general population. In addition, the EFA revealed that only half the items loaded on the expected factors. As a consequence of these unsatisfactory results of the original ECR-S12 with 12 items, a short form ECR-S6 with six items was construed and tested psychometrically.

Even though the ECR-S12 in the present representative sample showed only mediocre reliability values, the new ECR-S6, in contrast, showed similarly good reliability values as the ECR-S12 published in the literature [17] as well as the long version [24]. Therefore, the shortening process improved the reliability of this ECR-S6 version. Furthermore, factorial validity could be shown by CFA. An additional test for measurement invariance of the ECR-S6 revealed scalar invariance across gender and age.

The sociodemograpgic specificities of the ECR showed women reported higher values on the attachment anxiety scale than men, with no differences for the attachment avoidance scale. These findings of higher attachment anxiety in females than in males (meta-analysis, [25]) is also in line with the literature. Also, the higher attachment anxiety in females could recently be replicated in a large representative sample [26]. However, the present results on the ECR did not replicate the lower avoidant attachement in females compared to males of the meta-analysis [25]. Concerning the relationship status, the participants living together reported lower scores on the attachment anxiety and avoidance scale than participants not living together. This is in line with results on the legal relationship status. Hereby, married individuals were more securely attached versus unmarried individuals who were more insecurely attached [27, 28, 29]. Concerning the influence of education on attachment, no differences were found between different levels of education for attachment anxiety. However, individuals with an educational level of ≤ 8 years reported significantly higher attachment avoidance levels compared to the group with 9–11 years of education. These results replicate the results by Petrowski et al. [21], however, they contradict the findings by Bakermans-Kranenburg et al. [30] using the AAI. For age, only a slight decrease in attachment anxiety could be observed with increasing age. This is in line with the results by Petrowski et al. [21]. Thus, the sociodemographic patterns of the ECR are in line with the results in the literature and show a good convergent validity of the ECR.

The strength of this study is the representativity of the general population sample. This large sample with a wide range in ages is ideal for testing the factorial structure of an instrument. However, there are some limitations. The large sample size might easily lead to small but significant correlation coefficients. The ECR-S12 is only a screening instrument which, in general, may possibly be unable to reflect the entire heterogeneity of the construct. The newly developed ECR-S6 needs to be validated further in independent samples and in respect to similar or other constructs. Additionally, results of longitudinal studies assessing the stability of the construct would be desirable as well as studies that focus on the cultural comparability of this test instrument.

In conclusion, the ECR-S6 is a reliable short instrument with a good factorial structure to assess the attachment of two closely related individuals. Due to its low number of items, it is most suitable for use in surveys of a variety of topics.

## Supporting information

S1 Data (SAV)
