# Correction: Revised short screening version of the attachment questionnaire for couples from the German general population

**DOI:** 10.1371/journal.pone.0234228

**Published:** 2020-05-29

**Authors:** Katja Petrowski, Elmar Brähler, Thomas Suslow, Markus Zenger

The first author, Katja Petrowski, is incorrectly noted as the corresponding author. The correct corresponding author is Markus Zenger. Markus Zenger’s email address is: markus.zenger@medizin.uni-leipzig.de.
